# Reimagining cardiac care with AI, LLMs, blockchain, and metaverse

**DOI:** 10.21542/gcsp.2026.9

**Published:** 2026-02-28

**Authors:** Ahmad Musamih, Khaled Salah, Mohammed Omar, Samer Ellaham

**Affiliations:** 1 Department of Management Science and Engineering, Abu Dhabi, UAE; 2 Department of Computer and Information Engineering, Abu Dhabi, UAE; 3 Heart and Vascular Institute, Cleveland Clinic Abu Dhabi, Abu Dhabi, UAE

## Abstract

**Objective:** Artificial intelligence (AI) is reshaping cardiology by analyzing complex data and enabling precision care. Among its recent advances, large language models (LLMs) have gained prominence for their potential to enhance clinical reasoning, documentation, and education. Alongside AI, blockchain, and metaverse-based extended reality (XR) are transforming data security, interoperability, and immersive visualization. This review consolidates the current literature on these technologies, evaluates their roles in cardiology, and introduces a conceptual framework for integrating them to support a secure, intelligent, and interactive digital ecosystem.

**Methods:** A structured narrative review was conducted using PubMed and Scopus to identify peer-reviewed studies published between 2018 and 2025. Systematic identification and screening of the literature were performed and reported using PRISMA 2020. Eligible publications included original research, reviews, and conceptual papers addressing the use of AI, LLMs, blockchain, or XR in cardiology. The literature was analyzed thematically, and findings were synthesized qualitatively to describe applications, limitations, and opportunities for integration.

**Results:** Across 50 studies, LLMs showed promise in documentation, education, and decision support but faced challenges in accuracy, validation, and bias. Blockchain applications improved data exchange, consent management, and provenance tracking, though scalability and interoperability remain unresolved. XR technologies enhanced procedural precision, training, and patient engagement but were limited by cost and validation. A conceptual integration model illustrates how these technologies function within a unified cardiovascular ecosystem where blockchain provides the trust layer, LLMs deliver analytical intelligence, and XR enables immersive interaction.

**Conclusion:** Integrating AI, LLMs, blockchain, and XR offers a pathway toward intelligent, secure, and interactive cardiovascular care. Future research should validate integrated frameworks, develop ethical and regulatory standards, and promote responsible adoption of AI in cardiology.

## Introduction

Cardiovascular disease remains the leading cause of death worldwide and continues to impose a growing clinical and societal burden^1,2^. Despite advances in diagnostics and therapeutics, traditional models of cardiac care that rely on episodic and clinic-based assessment are increasingly insufficient for the early detection and long-term management of major risk factors such as hypertension, dyslipidemia, diabetes, and arrhythmias^3–5^. Digital health technologies, including wearable biosensors, remote monitoring systems, AI-supported imaging, and algorithm-driven decision support tools, have emerged as important innovations capable of extending cardiac care beyond in-person encounters^6–8^. These technologies offer opportunities to identify disease earlier, personalize treatment strategies, and support continuous monitoring of high-risk individuals^9,10^. However, the adoption and benefits of digital cardiovascular tools vary significantly across regions and health systems, which highlights the need for rigorous evaluation, thoughtful integration into routine workflows, and equitable access to ensure that digital health contributes meaningfully to reducing the global burden of cardiovascular disease^11^.

Building on these trends, cardiology has emerged as one of the specialties most profoundly reshaped by the broader digital transformation of healthcare. The field generates vast, diverse data streams from imaging, electrophysiology, genomics, and wearable sensors, making it uniquely suited for digital innovation. Over the past decade, research in cardiovascular medicine has expanded rapidly, moving from early work in telemedicine and remote monitoring to more advanced applications of artificial intelligence (AI), multimodal wearables, and integrated mobile health ecosystems^12^. These advances have improved access to care, enhanced continuous patient monitoring, and enabled real-time data sharing across health systems. At the same time, they have introduced new challenges related to data volume, heterogeneity, and interoperability, as cardiovascular information now spans multiple modalities, clinical environments, and device platforms^13^.

Recent literature emphasizes that the future of cardiology will depend on the ability to integrate these fragmented data sources into cohesive, intelligent systems. Emerging frameworks such as digitalomics, which combines molecular, imaging, and personal health data, and digital twin models, which simulate patient-specific cardiac physiology, represent early steps toward this vision^14^. Mobile health applications and connected devices have already demonstrated potential in risk factor management, patient education, and adherence monitoring, but they also highlight disparities in access and the need for standardized data governance^15^. The field is therefore shifting from basic digitization toward genuine digital transformation, where AI, machine learning, and interconnected health records collectively redefine cardiovascular care. As digital health tools become embedded within clinical workflows, the focus is moving from episodic encounters to continuous, data-driven, and patient-centered management of cardiovascular disease^16^. To support clarity and ensure consistent use of terminology in the sections that follow, Table 1 summarizes the core digital technologies discussed in this review and outlines their relevance to cardiovascular care.

**Table 1:** Key digital technologies and their relevance to cardiovascular care.

**Term**	**Definition**	**Relevance to cardiology**
**Artificial Intelligence (AI)**	A field of computer science that enables algorithms to perform tasks associated with human cognition, such as pattern recognition, classification, prediction, and natural language processing^17^.	AI supports automated ECG and imaging interpretation, prediction of clinical deterioration, diagnostic decision support, and integration of multimodal cardiovascular data.
**Large Language Models (LLMs)**	A category of AI models trained on very large text corpora that can summarize information, interpret unstructured text, generate new text, and respond to questions in a context-aware manner^18^.	LLMs assist with clinical documentation, drafting reports, summarizing cardiology guidelines, extracting structured information from electronic health records, and facilitating patient communication.
**Blockchain**	A distributed and cryptographically secured ledger that records data across multiple nodes so that entries are transparent, traceable, and resistant to modification^19,20^.	Blockchain enables secure exchange of ECG and imaging data, supports patient-controlled consent and identity management, and provides reliable provenance for datasets used to train AI models.
**Metaverse and Extended Reality (XR)**	XR refers to virtual, augmented, and mixed reality environments that enable users to interact with digital objects in 3D. The metaverse is an interconnected virtual ecosystem where XR experiences can be shared, visualized, and integrated with real clinical data^21^.	XR enhances cardiac procedural planning, visualization of congenital heart disease, cardiac rehabilitation, surgical training, and remote collaboration. Metaverse platforms enable interactive simulation, digital twin integration, and shared virtual environments for education and team-based care.

AI has become the driving force behind the digital transformation of cardiovascular medicine, which enables data-driven diagnosis, risk prediction, and workflow optimization across clinical settings. Among its most recent advances, large language models (LLMs) have gained particular attention for their ability to interpret unstructured clinical data, automate documentation, and support communication within care teams. Alongside AI, blockchain, and extended reality (XR) technologies represent the most dynamic frontiers of this transformation, together advancing toward more intelligent, secure, and immersive cardiovascular care. Machine and deep learning models now interpret electrocardiograms, echocardiography, and cardiac imaging with near-expert precision, improving the detection of arrhythmias, heart failure, and coronary artery disease^22,23^. By integrating multimodal data from imaging, genomics, and wearable sensors, AI enables precision cardiology tailored to individual risk profiles. LLMs represent a distinct evolution of AI, extending these capabilities by automating clinical summaries, facilitating communication, and streamlining workflow integration^24,25^. Despite ongoing challenges with transparency and validation, AI continues to augment rather than replace clinical expertise, driving a shift toward proactive, data-driven cardiovascular care.

Blockchain technology complements this evolution by providing a secure and decentralized framework for managing cardiovascular data across interoperable systems. Through encrypted, tamper-resistant ledgers and smart contracts, blockchain allows patients to control access to their medical information while maintaining traceability and trust across institutions^26^. In cardiology, it enables the secure exchange of imaging and electrocardiographic data, supports telecardiology and wearable monitoring, and underpins reliable data pipelines for AI-driven analytics^27,28^. Hybrid systems combining blockchain with explainable AI have introduced transparent, patient-centric screening platforms that safeguard confidentiality while enhancing accountability. Although scalability and regulatory challenges remain, blockchain is emerging as a key enabler of trustworthy and interoperable digital cardiology infrastructures.

XR technologies, including virtual, augmented, and mixed reality, are similarly redefining cardiovascular practice. These immersive tools facilitate detailed three-dimensional visualization of cardiac anatomy, enabling clinicians to simulate interventions such as transcatheter valve replacement or congenital defect repair before performing them on patients^29^. They have become valuable in surgical planning, electrophysiology, rehabilitation, and medical education, offering safer training environments and improved procedural precision. Within the expanding metaverse ecosystem, virtual collaboration platforms allow cardiologists to conduct remote multidisciplinary consultations and real-time procedural guidance using holographic imaging and digital twins^30,31^. Integration with AI further enhances anatomical modelling, image segmentation, and procedural risk assessment, creating adaptive, patient-specific simulations. While issues related to standardization and ethical governance persist, these technologies collectively mark a transition toward a more intelligent, secure, and immersive era in cardiovascular medicine.

This review examines how emerging technologies are transforming cardiology, with particular attention to LLMs, blockchain, and XR within the metaverse. It synthesizes current evidence from the literature to describe how these innovations are being applied in cardiovascular diagnostics, therapeutics, education, and data management while assessing their reported benefits, limitations, and implementation challenges. Beyond summarizing published applications, the review identifies areas where research and clinical practice can evolve, highlighting opportunities for technological, clinical, and regulatory advancement. It also introduces a conceptual framework illustrating how these technologies can operate together and integrate with existing cardiovascular systems to create more intelligent, secure, and immersive models of care. By outlining the current state of knowledge and envisioning future directions, this review provides an evidence-informed and forward-looking understanding of how digital innovation can enhance precision, efficiency, and patient engagement in cardiovascular medicine.

## Methodology

This review uses a structured narrative approach to identify, evaluate, and summarize recent literature on the application of LLMs, blockchain, and metaverse-based XR technologies in cardiology. A systematic identification and screening process is employed, with transparent reporting of study selection using PRISMA 2020^32^.

### Data sources and search strategy

A literature search was performed in PubMed and Scopus to identify peer-reviewed studies published between 2018 and 2025. Parallel searches were conducted for each of the three technology domains using combinations of controlled vocabulary and free-text terms related to cardiology and cardiovascular medicine. Representative search strings included:

• (“large language model” OR “generative AI” OR “artificial intelligence” OR “machine learning” OR “natural language processing”) AND (“cardiology” OR “cardiovascular” OR “heart disease”)

• (“blockchain” OR “distributed ledger”) AND (“cardiology” OR “healthcare data”)

• (“metaverse” OR “virtual reality” OR “augmented reality” OR “extended reality”) AND (“cardiology” OR “cardiac care”)

Searches were restricted to English-language publications. Reference lists of key articles were reviewed to ensure comprehensive coverage.

### Inclusion and exclusion criteria

Studies were included if they:

1. Were peer-reviewed and published in English between 2018 and 2025.
2. Examined the application, evaluation, or conceptual discussion of LLMs, blockchain, or metaverse/XR technologies in cardiology or cardiovascular medicine.
3. Reported original research, systematic or narrative reviews, or technical studies with clear clinical or operational relevance.

Studies were excluded if they:

1. Were conference abstracts, non-peer-reviewed sources, or editorials without cardiology-specific relevance.
2. Addressed general healthcare technologies without a cardiology focus.
3. Represented technical or engineering studies without cardiology-specific clinical or operational relevance.
4. Were duplicate publications or secondary analyses of already included reviews.

### Study selection and data synthesis

**Figure 1.**
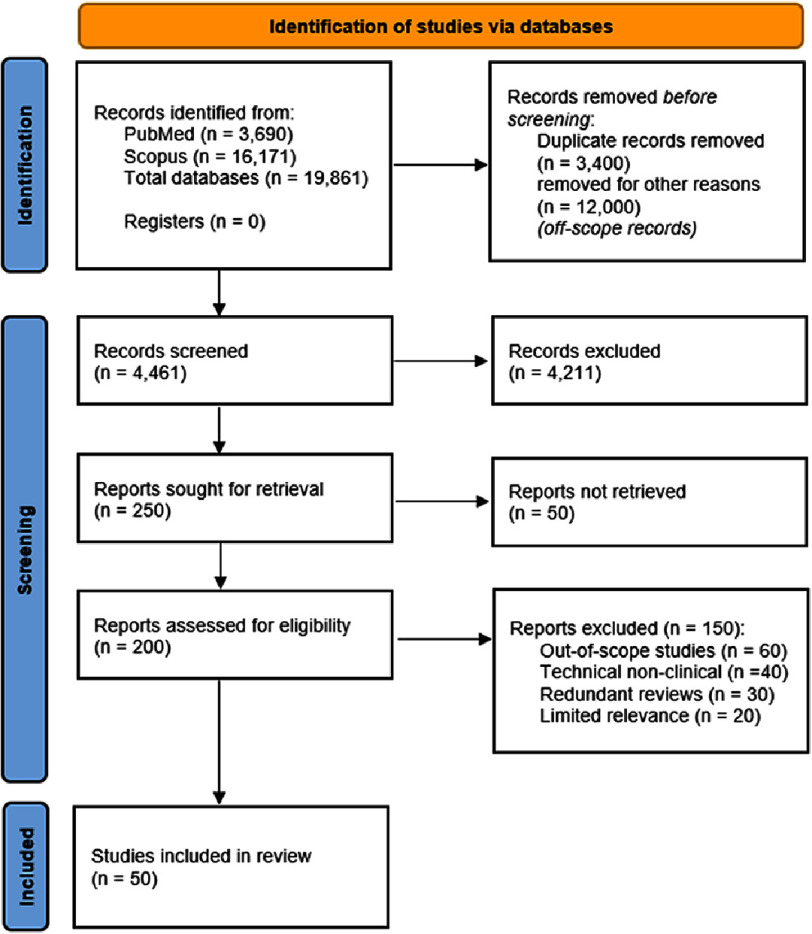
PRISMA 2020 flow diagram illustrating the identification, screening, eligibility assessment, and inclusion of studies in this review.

Following identification, duplicate records were removed. Given the intentionally broad search strategy, clearly non-cardiology or non-medical records were excluded during an initial pre-screening step prior to formal title and abstract screening. Remaining records underwent title and abstract screening, followed by full-text assessment for eligibility based on the predefined criteria. The complete study selection process is summarized in the PRISMA flow diagram Figure 1.

The included studies were grouped into three thematic domains: LLMs, Blockchain, and Metaverse/XR. Data extracted from each study included the technology type, clinical or operational application, study design, key findings, and reported challenges. Given the heterogeneity of study designs, outcomes, and maturity across domains, findings were synthesized qualitatively to identify current applications, benefits, limitations, and emerging research opportunities.

## Literature review

**Figure 2.**
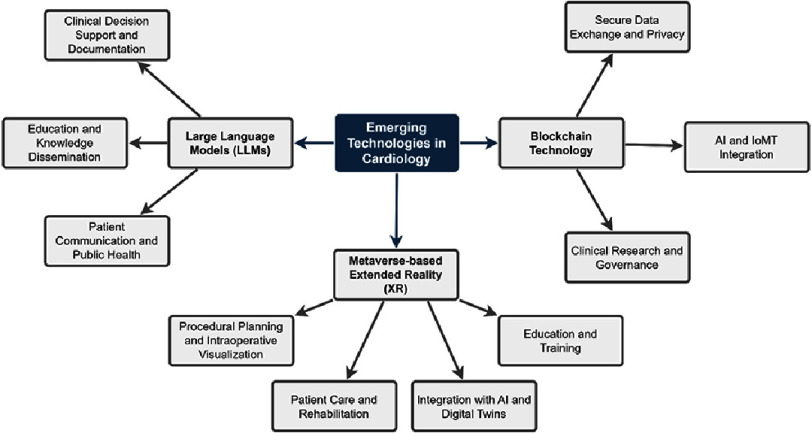
Key domains and applications of large language models, blockchain, and extended reality in cardiology.

The growing intersection between cardiology and digital innovation has led to a substantial body of research exploring how emerging technologies are reshaping cardiovascular care. Recent literature from 2018 to 2025 demonstrates increasing attention to LLMs, blockchain, and metaverse-based systems as potential tools to improve precision, efficiency, and patient engagement across the cardiology continuum. These technologies contribute to different yet complementary dimensions of modern cardiovascular practice, which encompasses intelligent automation, secure data management, and immersive learning or rehabilitation environments. Figure 2 presents a taxonomy summarizing the scope of these technological domains and their primary areas of application within cardiology. The following subsections summarize the published work on each technology, highlighting key applications, study findings, and common limitations reported in the literature.

### LLMs in cardiology

LLMs have rapidly emerged as one of the most dynamic innovations in cardiovascular medicine, with studies highlighting their expanding roles in clinical, educational, and administrative domains. The growing body of literature demonstrates both the potential of these models to augment cardiology workflows and the pressing need for validation, ethical governance, and domain-specific optimization.

#### Narrative and conceptual reviews of LLMs in cardiology

Bhattaru et al.^33^ reviewed early applications of LLMs in cardiology and outlined their capacity to automate language-based tasks, including clinical documentation, patient communication, and scientific writing. The authors reported moderate accuracy of LLMs in responding to cardiology-specific queries and interpreting guidelines, while emphasizing their potential to improve efficiency by reducing repetitive linguistic tasks. However, the study also highlighted key concerns, including hallucinations, biased data sources, and the lack of models trained on cardiology-specific datasets. The authors concluded that systematic validation and transparent reporting frameworks are essential before routine clinical adoption.

Ahmed et al.^34^ provided a comprehensive primer for cardiovascular healthcare professionals, explaining the operational principles of transformer-based LLMs and their applicability in everyday practice. They described how these models can summarise lengthy clinical notes, extract structured data, and assist with educational initiatives such as guideline interpretation and patient communication training. Reported advantages included improved access to evidence-based knowledge and reduced administrative burden. The authors cautioned, however, that widespread adoption remains constrained by issues of patient data security, misinformation, and limited real-world validation, and they recommended that LLMs be viewed as supportive tools operating under human supervision.

Building on this, Boonstra et al.^25^ presented a state-of-the-art review that positioned LLMs as integral components of the broader digital transformation of cardiology. Their paper detailed potential use cases ranging from documentation and billing automation to interactive chatbots for patient support. They proposed a conceptual framework for developing cardiology-specific LLMs capable of integrating text from electronic health records, imaging reports, and clinical literature to support diagnosis and research. In addition, they addressed technical and regulatory challenges such as data leakage, adversarial attacks, and the necessity of maintaining explainability. The review concluded that successful implementation of LLMs will depend on continuous monitoring, human oversight, and alignment with ethical and legal standards.

Sarraju et al.^35^ provided an overarching perspective on LLMs’ opportunities and challenges in cardiovascular medicine. The authors described their potential for generating clinical notes, transcribing patient encounters, and responding empathetically to patient questions, but cautioned against overreliance on unvalidated systems. They stressed that hallucinated data, outdated information, and algorithmic bias represent major risks and called for regulatory frameworks and clinician education to guide responsible adoption.

Wehbe^36^ provided a high-level commentary on the potential role of large language models in cardiology, spanning clinical care, education, and research. The article emphasized that current clinical applications of LLMs are concentrated in administrative and documentation support, such as clinical note generation, information extraction, and assistance with patient communication. Potential roles in decision support, risk prediction, and clinical reasoning were discussed, but the author cautioned that evidence remains limited and largely indirect. Key limitations highlighted included hallucinations, lack of up-to-date guideline awareness, inherited bias from training data, and the absence of prospective validation studies. The commentary underscored that meaningful adoption of LLMs in cardiology will require interdisciplinary collaboration, rigorous evaluation using clinical outcomes, and careful alignment with ethical and regulatory standards.

#### Empirical evaluations of LLMs in cardiology

Several empirical studies have evaluated LLMs in targeted cardiology applications. Nanda et al.^37^ assessed ChatGPT−3.5 using 360 questions from the Cath Self-Assessment Program (CathSAP) and found a significant performance improvement, from 54.4% to 79.2%, after the model was exposed to relevant educational material. The study demonstrated the model’s adaptability and its potential as an educational tool for cardiology trainees, while also identifying ongoing limitations in reasoning, spatial comprehension, and reliance on text-based data. Similarly, Itelman et al.^38^ evaluated commercially available LLMs for decision support in interventional cardiology. In this proof-of-concept study, ChatGPT-4 achieved perfect accuracy (100%) in determining appropriate management strategies between surgical and percutaneous interventions, outperforming ChatGPT−3.5 and Google Bard. The authors concluded that while modern LLMs show promise as adjuncts to multidisciplinary “heart-team” decision-making, challenges such as bias, data transparency, and outdated citation references must be resolved before clinical deployment.

Beyond clinical reasoning, recent research has also explored LLMs in automating cardiology documentation. Jung et al.^39^ fine-tuned an open-source Llama3.1-8B model using more than 17,000 real and synthetic discharge summaries to automate report generation for cardiac patients. The model achieved superior lexical accuracy and semantic coherence compared with other open-source LLMs, and expert cardiologists rated its outputs as clinically accurate and complete. These findings demonstrated that fine-tuned, domain-specific LLMs could improve workflow efficiency and documentation quality, though the authors emphasized the need for multi-institutional validation and standardized evaluation metrics.

Patient-facing applications are also being tested. Soundarrajan et al.^40^ compared ChatGPT−3.5 and Google Gemini in generating patient information guides for electrocardiography, echocardiography, and stress testing. Both models performed comparably in reliability, though Gemini produced more readable and accessible text, while ChatGPT achieved slightly higher reference alignment. The study underscored the potential of LLMs to improve public health communication and patient education but noted the importance of medical expert oversight to ensure accuracy and guideline compliance.

Van Veen et al.^41^ evaluated adapted large language models for clinical text summarization across multiple real-world documentation tasks, including radiology reports, progress notes, patient questions, and clinician–patient dialogue. Using a combination of quantitative natural language processing metrics and a blinded clinical reader study involving ten physicians, the authors found that adapted LLM summaries were rated as equivalent or superior to clinician-written summaries in most cases. Importantly, the study included a structured safety analysis that examined factual errors and potential clinical harm, showing that LLM-generated summaries produced fewer errors than medical experts in several categories. Despite these results, the authors emphasize that errors persisted in both human and model-generated summaries and that clinical deployment requires careful task selection, adaptation strategies, and ongoing oversight. This study provides strong evidence that LLMs can support documentation workflows, but it does not support autonomous clinical decision-making.

Tai-Seale et al.^42^ examined the integration of generative AI-drafted replies into electronic health record messaging workflows in a randomized quality improvement study involving primary care physicians. The use of AI-generated drafts was associated with longer message reading time and longer replies, while time spent composing replies did not decrease. Physicians reported that the drafts were useful as a starting point and helped improve tone and completeness, but still required review and editing. The study highlights that workflow integration of generative AI does not automatically translate into time savings and may introduce new cognitive steps into clinical communication. These findings underscore that the value of LLMs in clinical workflows may lie more in quality support and standardization than in efficiency gains, reinforcing the need for realistic expectations when deploying LLM-based tools in routine care.

#### Systematic evidence and practical guidance

Santos et al.^43^ conducted a systematic review of large language model applications in cardiovascular disease, following PRISMA guidelines. Thirty-five observational studies published between 2023 and 2024 were included, with most evaluating commercially available models, predominantly ChatGPT. Most studies focused on patient education and frequently asked questions related to cardiovascular risk factors and prevention, while a smaller proportion addressed clinical decision support. Across studies, LLMs generally produced accurate and appropriate responses, although omissions of guideline-directed therapies, occasional misinformation, and hallucinated references were reported. Importantly, all included studies were observational and most were rated as having a high risk of bias, with no randomized trials or outcome-based evaluations identified. The authors concluded that LLMs show promise as supplementary information tools for patients, but that evidence remains insufficient to support their use in individualized diagnosis or treatment decisions.

Nolin-Lapalme et al.^44^ provided a practical review of how large language models may be integrated into cardiovascular care and research workflows. The authors discussed applications such as summarizing medical records, extracting information from unstructured notes, supporting patient–physician communication, and assisting with literature review and data analysis. The review emphasized that general-purpose LLMs lack domain-specific knowledge required for independent clinical decision-making and require careful prompting, task selection, and validation. Key challenges identified included reasoning opacity, bias, context-window limitations, and uncertainty regarding generalizability across populations and institutions. The authors stressed that LLMs should currently be viewed as assistive tools that augment clinician workflows rather than autonomous systems, and that rigorous evaluation is required before broader clinical deployment.

#### LLMs within multimodal and task-specific cardiovascular AI systems

Ghaderi et al.^45^ reviewed the use of artificial intelligence for coronary artery calcification scoring, with a focus on deep learning and machine learning methods applied to CT-based imaging. The review summarizes evidence showing that AI-based systems can achieve high agreement with expert readers, with reported improvements in reproducibility and reductions in inter-observer variability. Several studies described strong performance metrics for automated CAC quantification across both ECG-gated and non-gated CT scans, supporting the feasibility of opportunistic screening from routine chest imaging. At the same time, the authors note that most available evidence is derived from retrospective analyses and technical validation studies rather than prospective outcome-driven trials. Important limitations include sensitivity to image quality, scanner heterogeneity, and training dataset composition, as well as reduced transparency of deep learning models that may affect clinician trust. The review concludes that AI-assisted CAC scoring is approaching clinical readiness for workflow support and risk stratification, but emphasizes the need for external validation across diverse populations and clearer evidence of impact on clinical decision-making and cardiovascular outcomes before widespread adoption.

Lin et al.^46^ reported a pragmatic, multicenter randomized clinical trial evaluating an AI-enabled electrocardiography alert system integrated into hospital workflows. In this study of more than 15,000 patients, real-time AI-ECG alerts delivered to treating physicians were associated with a statistically significant reduction in 90-day all-cause mortality, driven primarily by outcomes in patients identified as high risk by the model. The intervention prompted earlier escalation of care, increased diagnostic testing, and higher rates of intensive monitoring. While this trial represents one of the strongest examples of outcome-level benefit from AI deployment in cardiology, the system relied on a narrowly defined prediction task rather than language-based reasoning. The findings illustrate that clinically embedded AI tools can improve outcomes when tightly scoped, well integrated, and paired with clinician action, offering an important contrast to the current maturity of LLM-based systems.

Quer and Topol^47^ reviewed the role of large language models within the broader landscape of artificial intelligence in cardiovascular medicine, with particular emphasis on multimodal and foundation models. The authors described how transformer-based architectures can integrate text with imaging, biosensor data, genomics, and electronic health records to support diagnosis, risk prediction, and patient stratification. LLMs were positioned as a key interface layer that enables interpretation, summarization, and communication across complex multimodal systems rather than as standalone diagnostic tools. While promising applications were outlined, including workflow support and patient engagement, the authors emphasized unresolved risks related to privacy, data quality, bias, and clinical safety. The paper concluded that substantial validation and system-level integration are required before LLM-enabled multimodal AI can be adopted in routine cardiovascular care.

Jaltotage et al.^48^ reviewed the use of artificial intelligence in cardiovascular disease management, with particular emphasis on multimodal systems that integrate imaging, text, audio, and structured clinical data. The review outlined how multimodal AI may improve diagnostic accuracy, clinical decision support, and workflow efficiency compared with single-modality systems. However, the authors noted that truly multimodal implementations remain uncommon in routine cardiovascular care due to data heterogeneity, high computational requirements, and challenges related to system integration and validation. Although large language models were discussed as part of the broader AI ecosystem, their role was primarily described as supportive for handling unstructured data and facilitating workflow integration rather than direct clinical decision-making.

Acosta et al.^49^ provided a foundational review of multimodal biomedical artificial intelligence, describing how integrating diverse data types such as electronic health records, imaging, biosensors, and multiomics can enable more comprehensive representations of human health. The authors highlighted opportunities for personalized medicine, digital clinical trials, remote monitoring, and digital twin development, while also outlining key challenges related to data integration, privacy, and generalizability. Although not cardiology-specific, this work established the conceptual and technical basis for multimodal systems that later cardiovascular applications, including those incorporating large language models as text-processing interfaces, now build upon.

Lai et al.^50^ developed MAARS, a transformer-based multimodal artificial intelligence model to predict sudden cardiac death due to ventricular arrhythmias in patients with hypertrophic cardiomyopathy. The model integrated electronic health records, echocardiographic and radiology reports, and contrast-enhanced cardiac magnetic resonance images, achieving superior discrimination compared with established clinical risk tools across internal and external cohorts. MAARS demonstrated improved performance, fairness across demographic subgroups, and interpretable outputs through attention-based analyses. This study exemplifies how multimodal AI systems can outperform guideline-based risk stratification in high-risk cardiovascular populations, while also highlighting the contrast between task-specific, rigorously validated models and the current evidence base for general-purpose large language models.

Khera et al.^51^ presented a state-of-the-art review of artificial intelligence in cardiovascular care, covering applications across diagnostics, risk stratification, imaging, digital biomarkers, and clinical research. The authors emphasized the increasing role of multimodal and deep learning systems in extracting clinically meaningful patterns from unstructured data sources, including electrocardiograms, imaging, and electronic health records. Large language models were discussed within the broader AI landscape as tools that may assist with clinical documentation, information synthesis, and workflow support, rather than as standalone diagnostic systems. The review highlighted the need for rigorous validation, regulatory oversight, and equitable deployment to translate AI innovations from discovery into routine cardiovascular practice.

Ayoub et al.^52^ developed a multimodal fusion artificial intelligence model to predict immune checkpoint inhibitor–related myocarditis and major adverse cardiovascular events in cancer patients. The model combined baseline clinical data, laboratory values, and electrocardiogram signals in a joint fusion architecture, outperforming single-modality models for risk prediction. The study demonstrated the potential clinical utility of multimodal AI for early identification of high-risk patients who may benefit from closer cardiovascular surveillance. While not involving language-based reasoning, this work illustrates the maturity and clinical relevance of multimodal AI approaches that integrate heterogeneous cardiovascular data, providing an important benchmark against which emerging LLM-based tools can be contextualized.

Muse and Topol^53^ reviewed the application of multimodal artificial intelligence to cardiometabolic disease prevention and management, emphasizing the integration of clinical, imaging, genomic, and wearable data to enable personalized risk assessment and intervention. The authors described how transformer-based models facilitate self-supervised learning across heterogeneous data streams and support longitudinal risk prediction beyond traditional clinical calculators. Although the review did not focus on large language models specifically, it underscored the importance of multimodal frameworks in which LLMs may serve as interfaces for summarization, explanation, and patient engagement rather than as primary predictive engines.

Yang et al.^54^ reviewed the current state and future prospects of multimodal artificial intelligence in cardiovascular medicine, with emphasis on data fusion strategies, algorithmic frameworks, and clinical workflow integration. The review covered combinations of imaging, electrocardiography, electronic health records, multiomics, and wearable data, highlighting how multimodal AI can improve diagnostic accuracy and prognostic assessment. The authors noted that, despite promising results, challenges remain related to computational complexity, interpretability, data standardization, and real-world deployment. Large language models were positioned as components that may assist with unstructured data handling and clinical reasoning support within broader multimodal systems.

### Blockchain technology in cardiology

Blockchain applications in cardiology have evolved from conceptual frameworks to functional models that emphasize secure data sharing, diagnostic precision, and patient autonomy. Early efforts focused on using blockchain to address limitations in data interoperability and privacy that hinder the development of reliable AI systems for cardiovascular care. More recent studies have expanded this vision to include patient-centered frameworks, decentralized predictive models, and hybrid systems that combine blockchain with advanced analytics.

#### Conceptual foundations and early frameworks

Krittanawong et al.^26^ provided one of the earliest conceptual discussions on integrating blockchain with AI in cardiovascular medicine. They proposed blockchain as a decentralized infrastructure capable of linking fragmented health data sources, including electronic health records, wearable devices, imaging systems, and genomic repositories. The authors introduced the idea of an incentivized data ecosystem in which patients are rewarded for securely contributing anonymized information that supports AI model training and fairness. This framework positioned blockchain as a potential foundation for precision cardiology by enabling secure, traceable, and transparent information exchange between clinicians, researchers, and patients.

#### Blockchain-enabled diagnostics and screening systems

Building on these conceptual foundations, Borah et al.^27^ developed a blockchain-enabled HeartCare framework for diagnosing cardiovascular diseases through electrocardiogram signal analysis on resource-constrained devices. Their system incorporated a lightweight binary neural network to classify ECG signals, while blockchain ensured decentralized data storage and patient-controlled access. The integration of InterPlanetary File System storage (IPFS) improved scalability for large datasets, and validation on global ECG databases achieved an accuracy of 95.93%. This study demonstrated how blockchain could move beyond theory to serve as a secure backbone for telecardiology and continuous cardiac monitoring.

Muneer et al.^28^ extended blockchain’s use in diagnostics through a chatbot- based cardiovascular screening system powered by explainable artificial intelligence. Their approach combined blockchain for decentralized data security with explainable artificial intelligence to ensure transparency and interpretability in automated predictions. Using structured clinical data such as blood pressure, cholesterol, and glucose levels, the system achieved a diagnostic accuracy of 97.12%, surpassing conventional models. This integration highlighted blockchain’s dual role in safeguarding data and ensuring accountability in AI-driven cardiology diagnostics.

#### Secure predictive modeling and decentralized analytics

As blockchain matured as a health-data management technology, several studies explored its role in enhancing data integrity and governance within predictive and educational frameworks. Ahmed et al.^55^ implemented a private Hyperledger Fabric blockchain with cryptographic encryption to ensure secure data storage for heart-disease prediction. Using multiple classifiers and ensemble learning, their system achieved 89.2% accuracy and reinforced blockchain’s value in maintaining patient data ownership and privacy. Similarly, Otoum et al.^56^ combined blockchain with federated learning to enable collaborative model training across multiple healthcare institutions. The blockchain ledger was used to record model updates and enforce coordination through smart contracts, achieving 82.2% accuracy while preserving data confidentiality. Together, these studies demonstrated that blockchain can facilitate decentralized analytics in cardiology, supporting both predictive modeling and multi-institutional collaboration.

The integration of blockchain with machine learning for predictive analytics has further advanced its relevance in cardiovascular medicine. Desai and Basavarajaiah^57^ developed a hybrid framework combining Ethereum smart contracts with off-chain IPFS storage to secure electronic medical records while supporting disease prediction through machine-learning classifiers. Using XGBoost and Random Forest models trained on large datasets, they achieved accuracies ranging from 88% to 96%. Blockchain ensured patient authorization, immutable audit trails, and protection against data breaches, confirming its value as a decentralized infrastructure for predictive cardiovascular analytics.

#### Patient identity, data ownership, and governance models

Parallel research has also explored blockchain’s potential for redefining patient engagement and digital identity management in cardiology. Bendayan et al.^58^ examined nonfungible tokens as blockchain-based digital assets for managing electronic health data. They described how nonfungible tokens could link diverse cardiology data sources, such as imaging, wearable, and hospital records, to individual patients, allowing data ownership and traceability across healthcare systems. The authors proposed new use cases for clinical trials and supply-chain verification of cardiac devices, envisioning a “cardioverse” where nonfungible tokens serve as secure digital identifiers for patients and providers within immersive virtual platforms.

#### Forward-looking and systems-level perspectives

De Novi et al.^59^ presented an editorial perspective on anticipated developments in blockchain-enabled healthcare, with particular attention to digital twins, artificial intelligence, and data governance. The authors argue that blockchain may play a central role in ensuring data provenance, auditability, and access control for complex AI-driven systems such as digital twins, especially in settings that rely on large volumes of longitudinal patient data. Proposed applications include clinical trials, precision public health, and patient-centric data ownership models. However, the paper is primarily forward-looking and conceptual in nature, without cardiology-specific validation studies or empirical evidence demonstrating clinical impact. Many of the proposed use cases, including blockchain-secured digital twins and generative AI companions, remain speculative and depend on unresolved challenges related to scalability, regulation, interoperability, and ethical governance. Within cardiology, this work is best interpreted as outlining a possible future role for blockchain as an enabling infrastructure for trustworthy data management rather than as evidence of current clinical readiness or deployment.

Sowan et al.^60^ proposed a comprehensive conceptual framework for integrating metaverse environments with explainable artificial intelligence and blockchain to support healthcare delivery, education, and operations. The framework describes virtual consultation spaces, immersive training environments, digital twins for scenario modeling, and blockchain-based identity and data governance mechanisms. Explainable artificial intelligence techniques are incorporated to increase transparency of model outputs, while blockchain is used to provide immutable audit trails, consent management, and secure data exchange. The authors report pilot deployments across urban hospitals, rural clinics, and academic institutions to assess feasibility, scalability, and user acceptance, although these evaluations remain descriptive rather than outcome driven. The work is not cardiology-specific and does not include clinical validation in cardiovascular populations. Instead, it serves as a systems-level blueprint that illustrates how blockchain and immersive environments could support trustworthy, interoperable healthcare infrastructures, with potential relevance to cardiology workflows that rely on complex multimodal data and longitudinal care.

Sonkamble et al.^61^ described a blockchain-secured metaverse framework for telemedicine that combines immersive virtual environments, decentralized data management, and AI-supported analytics. The proposed system includes avatar-based virtual consultations, smart contract–driven consent and access control, off-chain encrypted storage of health records, and blockchain-based auditability to support regulatory compliance. Performance evaluations focused on technical metrics such as latency, scalability, transaction throughput, and user satisfaction, with reported improvements compared to traditional centralized telemedicine platforms. While the framework demonstrates technical feasibility and addresses challenges related to data security, interoperability, and patient engagement, it does not present disease-specific clinical outcomes or cardiology-focused validation. As such, the study contributes primarily to the infrastructure and governance literature, illustrating how blockchain and metaverse technologies could enable secure and scalable telemedicine platforms that may later be adapted for cardiovascular care.

### Metaverse and Extended Reality in Cardiology

The use of XR technologies, encompassing virtual, augmented, and mixed reality, has gained significant attention in cardiology as a means to enhance visualization, education, and procedural precision. Recent research has expanded the role of XR from simulation-based learning to clinical decision-making and patient engagement, while also exploring its integration with AI and the metaverse ecosystem.

### Foundational reviews and early XR applications

Early reviews established the foundation for XR adoption in cardiovascular medicine. Silva et al.^62^ described how advances in mobile computing and sensor technologies made XR tools viable for education, pre-procedural planning, intraprocedural visualization, and rehabilitation. Their review detailed how mixed reality systems, such as the Enhanced Electrophysiology Visualization and Interaction System (ELVIS), enable clinicians to manipulate 3D data in sterile environments, while holographic systems, such as RealView, provide real-time intracardiac visualization. These applications demonstrated how immersive visualization bridges the gap between physical and digital data to improve accuracy and interactivity in cardiovascular care.

### XR across cardiology subspecialties and workflows

The breadth of XR use across cardiology subspecialties has been well documented. Minga et al.^29^ reviewed applications of virtual and augmented reality in interventional, structural, congenital, and electrophysiology domains. The authors explained that XR enables accurate device positioning and procedural rehearsal for transcatheter valve replacement, left atrial appendage occlusion, and septal defect closure. In heart failure management, it assists in ventricular assist device planning, and in electrophysiology, it enhances spatial mapping during ablation. They also reported measurable improvements in workflow efficiency, including shorter procedures and reduced radiation exposure. Similarly, Jung et al.^63^ described how VR and AR improve visualization during transcatheter valve replacement and mitral repair by combining multiple imaging modalities into shared 3D views. Their review also highlighted patient-facing applications, such as VR-based rehabilitation programs that enhance recovery and lower anxiety levels. Together, these studies underscore XR’s dual benefits for clinicians and patients through enhanced spatial understanding, educational value, and therapeutic engagement.

### Congenital and structural cardiology applications

As XR systems became more sophisticated, their applications extended to congenital and structural cardiology. Goo et al.^64^ demonstrated that CT and MRI data can be reconstructed into interactive 3D models for preprocedural planning in congenital heart disease. Augmented and mixed-reality overlays improved surgical navigation and helped physicians visualize cardiac anatomy intraoperatively, while VR simulations supported training in rare congenital morphologies. Annabestani et al.^65^ further expanded on interventional applications, describing how AR and MR systems integrate imaging modalities such as fluoroscopy and ultrasound to produce holographic overlays that assist with catheter navigation and spatial orientation. Clinical examples included the Enhanced Interaction Electrophysiology Visualization System, which improved navigation accuracy by one-third compared with conventional methods, and MR headsets that reduced catheterization times by roughly 20%. These findings demonstrate XR’s ability to merge real-time imaging with procedural guidance to improve precision and safety.

### Educational, collaborative, and metaverse-oriented frameworks

The educational and collaborative dimensions of XR continue to evolve toward more immersive digital ecosystems. Skalidis et al. ^66^ introduced the concept of the “CardioVerse”, in which VR, AR, and blockchain converge to create a metaverse environment for cardiovascular medicine. They proposed virtual clinics where patients and clinicians interact through avatars, using real-time physiological data such as ECG and blood pressure. The paper highlighted how global collaboration could be facilitated through virtual surgical observation and interactive training modules. Building on this, Tsai et al.^67^ defined XR as a comprehensive continuum linking physical and digital learning spaces across cardiovascular care. Their review showcased educational platforms like Stanford’s Virtual Heart and Case Western’s HoloAnatomy, where trainees can manipulate 3D cardiac structures interactively. Both studies emphasized that these immersive approaches foster engagement, understanding, and collaboration across geographic boundaries.

### Clinical planning, rehabilitation, and procedural efficiency

Beyond education, XR technologies have demonstrated clinical relevance in diagnostic and therapeutic planning. Rashidova et al.^30^ analyzed 55 studies on VR and AR in cardiology and cardiac surgery, showing consistent benefits for preprocedural planning, medical education, and rehabilitation. They reported that VR-based preoperative simulations improve surgical precision and reduce anxiety among patients and clinicians. Moon et al.^68^ elaborated on AR’s contribution to procedural efficiency, describing how holographic overlays derived from CT, MRI, and echocardiography enhance visualization in coronary interventions, transcatheter aortic valve replacement, and electrophysiology mapping. These systems not only reduce procedure time and contrast use but also support clinician training by simplifying complex anatomical concepts. Ismail Sharieff et al.^69^ complemented these findings by summarizing innovations in holographic displays and AR headsets for intraoperative guidance, education, and patient rehabilitation. They noted that immersive rehabilitation programs promote post-event exercise adherence and recovery, highlighting XR’s potential across the entire continuum of cardiac care.

### Integration with AI and digital-twin technologies

Recent advances have integrated XR with AI and digital-twin technology to personalize cardiology further. Rudnicka et al.^70^ reviewed 253 studies on health digital twins, emphasizing how XR environments combined with AI segmentation algorithms can automatically delineate cardiac lesions and optimize therapy planning. They also described touch-free XR interfaces using gesture and voice control, which enhance usability in sterile operating environments. Skalidis et al.^31^ similarly reported that combining AI with XR technologies improves diagnostic precision, reduces observer variability, and enables virtual rehearsal of transcatheter aortic valve replacement procedures. These developments represent the transition of XR from an educational tool to a key component of precision medicine and individualized treatment design.

### Systematic reviews and evidence maturity

The scope and evidence base for XR in cardiovascular medicine have been further consolidated through large-scale systematic reviews. Kanschik et al.^71^ analyzed 164 peer-reviewed studies, categorizing XR applications into eight cardiology domains ranging from valvular and congenital heart disease to cardiac rehabilitation. Their findings showed that XR use leads to shorter procedures, reduced radiation exposure, improved diagnostic accuracy, and better patient satisfaction. They also identified imbalances in evidence, noting that most studies focused on VR, while augmented and mixed-reality applications remain underrepresented. Sun and Vaccarezza^72^ summarized and contextualized these findings, emphasizing that XR has already demonstrated significant educational, procedural, and rehabilitative benefits but that larger randomized controlled trials are required to establish its long-term clinical value.

AbdelMassih et al.^73^ presented an umbrella review examining the use of virtual reality and artificial intelligence in cardiac interventions and cardiac surgery, with a particular emphasis on pediatric and congenital heart disease. The review synthesized three prior reviews comprising 71 studies and showed that immersive VR has been used most often in cardiac surgical training, especially for mitral valve and coronary procedures. Use of VR for procedural planning was less frequent and was largely limited to small case series and feasibility reports. Across the included studies, VR was associated with improved anatomical understanding and increased surgeon confidence, but outcomes were mainly subjective and surrogate in nature. No consistent evidence was provided to demonstrate improvements in procedural efficiency or patient-level cardiovascular outcomes. The authors also noted practical limitations related to reliance on CT and CMR imaging for 3D reconstruction, which raises concerns about radiation exposure, cost, and scalability beyond highly specialized centers. Artificial intelligence was discussed primarily in relation to conventional machine learning and convolutional neural network approaches for imaging analysis and outcome prediction rather than language-based or multimodal foundation models. Overall, this review indicates that XR technologies in cardiology are supported mainly by pilot and feasibility-level evidence and currently appear most appropriate for education and selected planning applications rather than routine high-risk clinical use.

### Acute care and global equity perspectives

Bruno et al.^74^ provided an editorial overview of emerging applications of virtual reality in acute cardiovascular care, focusing on real-world clinical contexts rather than experimental simulation alone. The authors discussed the use of VR for post-discharge heart failure management, pre-procedural planning, and patient education, drawing on recent studies that evaluated VR in inpatient and peri-procedural workflows. Reported benefits included improved spatial understanding during pre-procedural planning, reduced patient anxiety before cardiac surgery, and potential support for post-care optimization. At the same time, the editorial emphasized that most current applications remain limited to pilot studies and small clinical evaluations, with inconsistent evidence for effects on hard outcomes such as mortality or rehospitalization. The authors also highlighted barriers to routine adoption, including lack of standardized evaluation frameworks, limited integration with hospital systems, and restricted availability of AI-driven analytics outside academic centers. Overall, this editorial positions VR as a supportive clinical tool with emerging workflow relevance, while underscoring that evidence for routine use in acute cardiovascular care remains preliminary.

Shrestha et al.^75^ reviewed the use of virtual and augmented reality in cardiovascular care within low- and middle-income countries, with emphasis on clinical implementation rather than technical development alone. The review covered applications across cardiac rehabilitation, patient education, medical training, and procedural planning, citing small clinical trials and pilot studies that reported reductions in patient anxiety, improved rehabilitation adherence, and enhanced procedural understanding. The authors noted that most available evidence originates from small studies with heterogeneous designs and limited follow-up, often conducted in middle-income rather than low-income settings. Major barriers to implementation included hardware cost, infrastructure requirements, lack of trained personnel, and limited institutional funding. The review also highlighted challenges related to patient acceptance, digital literacy, and the absence of supportive policies. While VR and AR were presented as potentially scalable tools for extending cardiovascular care and training in resource-constrained environments, the authors concluded that broader adoption would depend on cost reduction, simplified platforms, and stronger evidence from larger, context-specific clinical studies.

### Research gap

Table 2 provides a comparative summary of findings from the reviewed literature, which consolidates the main applications, demonstrated benefits, limitations, and integration potential of LLMs, blockchain technology, and metaverse-based systems in cardiology. Although each of these technologies has advanced specific aspects of cardiovascular care, the current literature indicates that their development has taken place in parallel rather than through coordinated or interoperable efforts. Literature consistently highlights the individual strengths of these technologies, with LLMs supporting automation and clinical communication, blockchain ensuring security and data integrity, and XR enhancing visualization, education, and patient engagement. However, few studies have examined how these systems might work together to form a unified digital framework for cardiology.

**Table 2:** Comparative overview of large language models, blockchain, and metaverse-based technologies in cardiology.

**Technology**	**Primary Applications**	**Demonstrated Benefits**	**Current Limitations**	**Potential for Integration with Other Technologies**
**LLMs**	• Clinical documentation and report summarization • Decision support and guideline interpretation • Patient communication and educational chatbots • Scientific writing and literature synthesis	• Reduces administrative workload and enhances workflow efficiency • Improves accessibility to evidence-based information • Enables interactive education and communication	• Hallucinations and factual inaccuracies • Lack of cardiology-specific fine-tuning and validation • Privacy and data-security concerns • Limited real-world clinical evidence	• Can serve as the analytic layer for blockchain-secured data • Can power metaverse interfaces through conversational agents and real-time translation
**Blockchain**	• Secure sharing of ECG, EHR, and imaging data • Decentralized patient-consent and identity management • Smart contracts for clinical trials and data provenance • Integration with AI and IoMT for predictive modeling	• Enhances data integrity, transparency, and traceability • Empowers patient data ownership • Enables cross-institutional collaboration • Supports explainable and auditable AI models	• Scalability and energy-efficiency challenges • Lack of standardized regulation and interoperability • Implementation cost and technical complexity	• Provides the trust layer for LLM- or AI-based analytics • Can underpin secure metaverse ecosystems and digital identity management
**Metaverse and Extended Reality (XR)**	• Procedural planning and intraoperative visualization • Education, simulation, and global collaboration • Patient rehabilitation and anxiety reduction • Integration with AI and digital-twin modeling	• Improves spatial understanding and procedural precision • Enhances engagement for trainees and patients • Reduces procedure time, radiation exposure, and anxiety • Supports personalized and immersive care	• High implementation cost and equipment limitations • Absence of large, randomized trials • Data-privacy and connectivity concerns • Technical standardization required	• Offers the immersive front end for LLM-driven or blockchain-secured cardiology systems • Can integrate with AI and digital twins for precision-guided interventions

Research on LLMs has focused mainly on text generation, clinical documentation, and educational applications, while their integration with real-time clinical data remains largely conceptual. Blockchain studies have demonstrated secure data management and provenance tracking but rarely explore the feasibility of embedding intelligent or interactive analytics within these frameworks. Similarly, work on XR technologies has centered on immersive training and procedural visualization without connecting these systems to secure data networks or decision-support models.

Across all three domains, the literature reveals overlapping limitations that prevent full clinical translation. Fragmented infrastructures, inconsistent evaluation criteria, and limited multi-institutional validation remain common challenges. Ethical, legal, and governance mechanisms to guide the responsible integration of these technologies are also lacking, creating uncertainty about accountability, bias management, and data ownership when they are used together.

These gaps indicate a clear need for a more integrated research approach that aligns technological innovation with clinical practicality. Bringing together LLMs, blockchain, and XR under a shared cardiology framework could enable intelligent, secure, and immersive systems. Such integration has the potential to transform cardiovascular care by combining precise analytics, trustworthy data management, and interactive patient engagement.

## Framework for the integration of LLMs, blockchain, and XR in cardiology

**Figure 3.**
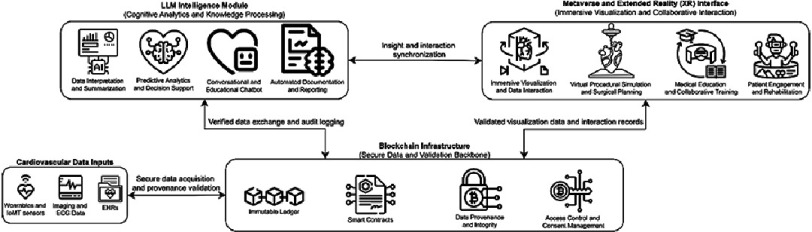
Theoretical Integrated framework of large language models, blockchain, and metaverse technologies in cardiology.

The convergence of LLMs, blockchain, and metaverse-based XR represents a pivotal opportunity to transform the digital landscape of cardiovascular care. Each of these technologies has demonstrated independent value across diagnostic, educational, and data management domains, but their true potential lies in their integration into a cohesive, interoperable ecosystem. Figure 3 illustrates a theoretical framework for such integration, showing how secure data management, intelligent analytics, and immersive interaction operate together to enhance precision, security, and patient engagement throughout the cardiology domain.

 At the foundation of this framework, blockchain infrastructure functions as the secure data and validation backbone. It enables the immutable recording of cardiovascular data, including imaging, electrocardiograms, and wearable-derived metrics, while managing consent and provenance through smart contracts. This layer guarantees the integrity, transparency, and accountability of all data transactions across the system. The blockchain verifies incoming data from electronic health records (EHRs) and Internet of Medical Things (IoMT) devices and provides an auditable trail for every analytic and user interaction within the ecosystem.

Building upon this foundation, the LLM intelligence module acts as the cognitive processing engine of the framework. Using verified datasets retrieved from the blockchain, LLMs interpret and synthesize multimodal cardiovascular information into clinically meaningful insights. As shown in Figure 3, this module performs core analytical functions such as data summarization, predictive modeling, and automated documentation, while also enabling conversational and educational interfaces. Bidirectional interaction between the blockchain and LLM modules allows analytic outputs, documentation, and audit logs to be securely recorded, which ensures transparency and facilitating model traceability.

Parallel to the LLM intelligence module, the metaverse and XR interface provides the experiential and collaborative dimension of the system. This component translates AI-driven insights into immersive and interactive visualizations that support clinical decision-making, medical training, and patient engagement. Within this environment, validated cardiovascular data are rendered into dynamic three-dimensional representations that clinicians and trainees can manipulate for procedural planning, while patients can engage in guided rehabilitation or educational sessions. As indicated in Figure 3, continuous data verification between the XR interface and blockchain infrastructure ensures that all displayed assets and interactions are authentic and securely logged.

A central element of this integration is the synchronization between the LLM and XR modules, which enables seamless communication between the analytical and experiential domains. LLMs supply the metaverse interface with natural language explanations, predictive outputs, and adaptive dialogue for clinical or educational use. In return, data generated from XR-based simulations, user feedback, and patient interactions can inform subsequent analytic refinements within the LLM environment. This continuous feedback loop creates an intelligent, secure, and interactive cardiology ecosystem capable of evolving through real-world application.

This proposed architecture establishes a framework for next-generation cardiovascular care. By combining the trust of blockchain, the intelligence of LLMs, and the interactivity of XR, it supports a future model of cardiology that is transparent, data-driven, and patient-centered. Beyond technical interoperability, such integration emphasizes ethical accountability, standardization, and equitable access, which are essential for the responsible adoption of emerging technologies in clinical cardiology.

## Applications for the Integrated Framework in Cardiology

**Figure 4.**
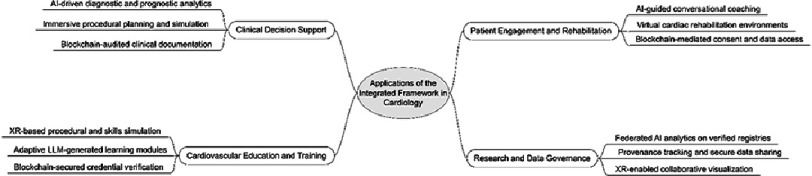
Taxonomy of Applications of the Integrated Digital Framework in Cardiology.

In this section, the potential applications arising from the integration of LLMs, blockchain, and XR are explored. The framework proposed in this review enables secure data provenance, intelligent analytics, and immersive interaction within a unified digital ecosystem. Figure 4 presents a taxonomy of these applications, organized into four primary domains comprising clinical decision support, cardiovascular education and training, patient engagement and rehabilitation, and research and data governance. These domains represent areas where the combined functionality of the three technologies offers advantages that cannot be achieved through their independent use.

### Clinical decision support

The integration of LLMs, blockchain, and XR can transform decision support in cardiology by combining analytic reasoning, data integrity, and spatial understanding. LLMs can generate real-time diagnostic and prognostic analyses from verified multimodal data, including imaging, electrocardiography, and clinical documentation. Blockchain ensures the authenticity and traceability of these inputs, while recording the analytic outputs for quality assurance and future audit. XR environments allow clinicians to interact with patient-specific visualizations, such as reconstructed cardiac anatomy with annotated risk scores or procedural guidance overlays. Together, these capabilities enable transparent, verifiable, and context-aware decision-making that enhances both efficiency and accountability.

This approach directly addresses the fragmentation of digital data in cardiovascular care, where imaging, hemodynamic, and clinical information often remain siloed. Integrating these sources through a secure, AI-driven workflow could streamline preprocedural conferences, reduce duplication of testing, and facilitate guideline-concordant decisions. Future development should focus on integrating real-time LLM reasoning into clinical decision support systems already approved for use in cardiology, evaluating how blockchain-based provenance tracking influences clinician confidence and diagnostic accuracy in prospective trials.

### Cardiovascular education and training

Simulation-based education and skill development can benefit substantially from the proposed framework. XR offers immersive environments for procedural rehearsal, which allows trainees to practice catheter placement, valve deployment, or echocardiographic interpretation with immediate visual feedback. LLMs can adapt educational content to a learner’s proficiency level, generate interactive explanations, and provide real-time performance insights. Blockchain adds an essential layer of verification by maintaining secure learner profiles, recording credentialing activities, and ensuring the authenticity of educational materials. This integration supports a more standardized, traceable, and adaptive training ecosystem for cardiovascular professionals.

Such an ecosystem can also help address current gaps in equitable access to procedural training, particularly in resource-limited settings where exposure to complex cases is infrequent. Blockchain-mediated credential portability could allow verified simulation records to accompany trainees across institutions, supporting international training equivalency. In the future, integrated LLM–XR training modules could contribute to competency-based education frameworks by objectively quantifying skill acquisition and retention while ensuring the authenticity of performance data.

### Patient engagement and rehabilitation

In patient-facing care, the integrated framework can foster greater engagement, adherence, and empowerment. LLMs can function as conversational health assistants that interpret wearable data, explain treatment plans, and promote adherence through personalized dialogue. Blockchain safeguards patient consent, manages access permissions, and ensures that data shared with clinicians or applications remain tamper-proof. XR platforms extend these capabilities into immersive cardiac rehabilitation and education programs, where patients can visualize heart function, monitor progress, and participate in guided virtual exercises. This triad of technologies creates a secure, interactive, and personalized environment that enhances patient understanding and participation in cardiovascular care.

This application directly supports the shift from episodic care to continuous, patient-managed health. By linking blockchain-verified data from wearables with conversational and immersive tools, clinicians could monitor adherence and progress in real time while preserving patient privacy. Future research should evaluate whether such systems improve long-term outcomes in secondary prevention and heart failure management, particularly in populations with limited access to conventional rehabilitation programs.

### Research and data governance

Multi-institutional cardiovascular research and data governance stand to gain from the synergistic use of these technologies. Blockchain enables transparent data provenance and consent management, which establishes trust across collaborative research networks. LLMs can analyze deidentified datasets distributed across blockchain-secured nodes, extracting insights without compromising privacy. XR can further enhance collaboration by providing shared virtual spaces for visualizing datasets, modeling trial results, and facilitating interdisciplinary discussion. The combination promotes reproducibility, interoperability, and ethical stewardship of data, which forms a foundation for the next generation of cardiology research and policy frameworks.

The integration also offers a pathway to harmonize global cardiovascular registries while maintaining jurisdictional data sovereignty. Decentralized data governance through blockchain could allow federated learning models to analyze outcomes across borders without transferring sensitive information. Future initiatives may focus on establishing standardized protocols for blockchain-mediated data exchange and developing interoperable XR research environments that support collaborative hypothesis generation and clinical trial visualization.

## Discussion and Future Directions

In this section, we interpret the findings of the reviewed literature, the proposed integration framework, and its practical applications to outline how emerging technologies can collectively advance cardiovascular medicine. The discussion synthesizes key patterns observed across studies, identifying how current progress in LLMs, blockchain, and XR can be leveraged to create a secure, intelligent, and interactive digital ecosystem. It also examines the barriers that continue to hinder implementation, the enablers that can facilitate adoption, and the broader implications for research, clinical practice, and policy. Finally, this section looks ahead to the evolving vision of digital cardiology, highlighting the ethical, technical, and organizational principles needed to translate this integrated model into real-world applications.

### Key findings and insights

The literature demonstrates steady progress within each technology domain, yet clinical impact remains uneven across settings and use cases. LLMs are advancing fastest in documentation, summarization, and education, with early signs of utility in decision support and exam performance, but external validation and reproducibility in multi-institutional cohorts are limited^76^. Blockchain pilots show credible advantages for provenance, consent, and auditability, though few studies quantify performance under realistic throughput, latency, and governance constraints across heterogeneous hospitals^77^. Metaverse and XR tools are maturing in procedural planning, congenital heart disease visualization, and rehabilitation, but evidence still concentrates in small trials and simulation environments rather than routine clinical pathways^60^.

Positioning blockchain as a secure backbone addresses a recurring weakness in both LLM and XR studies, namely uncertain data lineage and fragmented access control. When LLMs draw only from verified records and return their outputs to an auditable ledger, the resulting workflow is more transparent, traceable, and suitable for quality assurance. Likewise, XR scenes that render assets with recorded provenance can reduce ambiguity about which imaging series, segmentation, or digital twin parameters were used for a case. The synthesis suggests that the most credible near-term gains will emerge in tasks that are already documentation heavy and coordination heavy, such as preprocedural conferences, discharge planning, and longitudinal disease management, where secure data movement, summarization, and shared visualization directly align with clinical pain points^78^.

A second observation is that performance alone is not a sufficient predictor of adoption. Across studies, clinicians consistently emphasize reliability, clarity of responsibility, and workflow fit. LLM outputs that are accurate but hard to trace back to sources, XR tools that are compelling but poorly integrated into picture archiving systems, or blockchain ledgers that secure data but slow clinical access all face adoption headwinds. The integrated architecture directly targets these frictions by pairing verification first, analysis second, and visualization third, with bidirectional logging at each step. This sequence mirrors clinical expectations for documentation, safety checks, and multidisciplinary review, and can therefore increase trust even before randomized impact studies are completed^79^.

### Barriers and enablers of adoption

Several barriers recur across the literature and remain salient in an integrated deployment. Data heterogeneity limits consistent ingestion, especially when electrocardiography streams, echocardiography reports, and free-text notes follow different schemas. Interoperability standards such as HL7 FHIR and DICOM Structured Reporting are enablers but require disciplined implementation and conformance testing to be effective^80^. Model generalization is another barrier. LLMs fine-tuned on one institution’s style may drift or underperform elsewhere, especially if documentation templates, medication formularies, or language patterns differ. Federated learning and careful prompt standardization can help without centralizing raw patient data, particularly when paired with blockchain mediated consent and audit trails^81^.

Cost and maintainability are practical constraints. XR deployments need reliable rendering hardware, infection control procedures, and network bandwidth that does not interfere with clinical systems^61^. Blockchain brings operational overhead in node management, key custody, and smart contract updates, which must be absorbed by existing IT teams or a shared service. Clear ownership models and service-level targets are enablers, as are cloud managed options where appropriate governance permits^82^. Human factors are equally important. Cognitive load rises if clinicians must reconcile outputs from three tools. The proposed architecture reduces that load by allowing LLMs to translate verified data into concise, context aware summaries and by using XR to present only the task relevant elements of a case. Explicit interface guidelines, escalation rules, and documentation policies remain necessary to prevent alert fatigue and to define accountability when recommendations are accepted or overridden^83^.

Regulatory and ethical guardrails are not yet harmonized for cross-technology systems. Transparency requirements, data processing agreements, liability for model errors, and consent for derivative data used in simulation or education cannot be managed piecemeal. A unified governance approach is an enabler. It should couple role based access control, consent recording, dataset and model versioning, and incident response procedures, all observable through auditable logs on the ledger^84^.

### Implications for research, clinical practice, and policy

Research should shift from single technology performance reports to evaluations of end-to-end workflows anchored in the integrated model. Two priorities are prospective studies that measure time to decision, communication quality, and error detection in preprocedural meetings that combine LLM summarization with XR visualization, and pragmatic trials that instrument consent, provenance, and audit events on the ledger to quantify traceability benefits and downstream safety signals^85^. Methodologically, studies should report dataset lineage, prompt templates, error taxonomies, and human-in-the-loop review criteria and should release deidentified benchmarking bundles where feasible to enable replication. Shared evaluation targets, such as harmonized documentation quality metrics, provenance completeness scores, and XR task success measures, would allow cross site comparison and meta-analysis^86^.

For clinical practice, near-term integration opportunities include discharge summary generation with source-linked citations to the ledger, catheterization conference preparation where LLMs collate guideline-concordant summaries, and XR displays patient-specific anatomy, and rehabilitation programs that combine conversational coaching with verifiable progress logs. Implementation should follow staged deployment with shadow mode trials, differential access by role, and real-time monitoring of failure modes, supported by rollback plans and clear avenues for clinician feedback^87,88^. Training should prioritize three competencies. Clinicians need to read and question the rationales for models. Educators should design XR scenarios that map real documentation and device inventories. IT and compliance teams should operate and audit smart contracts, keys, and model versions. These competencies can be embedded in continuing professional development with simulation-based assessment^89^.

Policy should encourage safe data mobility and accountability rather than technology-specific mandates. Procurement can require support for established interoperability standards, human-readable audit trails, and patient accessible consent records. Reimbursement models may recognize documentation and coordination benefits where measurable reductions in time, duplication, or adverse events are demonstrated. Equity should be a design requirement. Institutions serving under-resourced populations may have limited capacity for XR hardware or for operating blockchain nodes. Shared infrastructure, regional nodes, and low-bandwidth XR modes can prevent widening disparities, while external validation should include multilingual documentation and varied health literacy levels^90^.

### Vision for the future of digital cardiology

The long-term goal is a reliable, seamless digital environment in which verification, interpretation, and visualization occur unobtrusively. In such a setting, a clinician enters a preprocedural review with a ledger-verified summary of the patient’s longitudinal record, a concise synthesis of key risks and options prepared by a domain-tuned language model, and an interactive scene that displays the anatomy and device plan aligned to the record of consent and prior decisions. Every step is logged to a shared, queryable ledger that supports quality improvement and research while preserving patient agency. Patients encounter the same coherence during rehabilitation and education, with understandable explanations, verified data linkages, and progress that follows them across institutions. Achieving this future requires careful engineering and governance, but the path is realistic. The components already exist in partial form, and the model shows how they can be arranged to work together. The remaining work is to demonstrate that such an arrangement is safe, efficient, and equitable at scale, and to refine it through continuous feedback from clinicians, patients, and system stewards^91,92^.

## Conclusion

In this review, we examined the growing influence of AI in cardiology, with a specific focus on LLMs, as well as blockchain- and metaverse-based XR. The paper proposed an integrated framework and outlined practical application domains, demonstrating how these technologies can work together within a unified digital ecosystem. It analyzed their combined use across cardiovascular diagnostics, therapeutics, education, patient engagement, and data governance while assessing their strengths, limitations, and readiness for clinical adoption.

The collective findings indicate that these technologies, although developed along separate paths, are complementary in purpose. LLMs represent the most transformative advancement within AI, providing the analytical and communicative intelligence to interpret cardiovascular data, while blockchain ensures data provenance and security, and metaverse-based XR enables immersive visualization and interaction. When connected through interoperable standards and ethical governance, these technologies can enhance precision, transparency, and collaboration across clinical and educational practice. The integrated model presented here highlights the potential of digital cardiology to advance from isolated technological innovation toward coordinated and patient-centered transformation.

Despite this potential, several limitations persist. The current literature is dominated by proof-of-concept studies with heterogeneous evaluation methods and small sample sizes. Technical and regulatory barriers including data fragmentation, scalability, cost, and the absence of harmonized governance frameworks continue to restrict widespread adoption. Future research should prioritize multi-institutional validation of integrated systems, the development of standardized performance benchmarks, and the creation of policies that promote interoperability, equity, and accountability. Achieving these goals will be essential for translating AI, particularly LLMs, along with blockchain and metaverse-based XR, from experimental innovation into reliable, ethical, and sustainable components of cardiovascular care.

## Author contributions

**Conceptualization:** Ahmad Musamih, Khaled Salah, Mohammed Omar, Samer Ellaham. **Methodology:** Ahmad Musamih, Khaled Salah, Mohammed Omar, Samer Ellaham. **Writing –Original Draft Preparation:** Ahmad Musamih. **Writing –Review & Editing:** Khaled Salah, Mohammed Omar, Samer Ellaham. **Investigation:** Ahmad Musamih. **Visualization:** Ahmad Musamih. **Supervision:** Khaled Salah, Mohammed Omar. **Project administration:** Mohammed Omar. **Fund Acquisition:** Mohammed Omar.

## Acknowledgments

This research was funded by the Socio-Technical Systems Lab (STSL), Khalifa University of Science and Technology (KU-STSL).

During the preparation and review of this work, the authors used ChatGPT to improve readability and language. After using this tool, the authors reviewed and edited the content as needed and took full responsibility for the content of the publication.

## Conflicts of interest

The author has no potential conflicts of interest to disclose.
